# ApmA Is a Unique Aminoglycoside Antibiotic Acetyltransferase That Inactivates Apramycin

**DOI:** 10.1128/mBio.02705-20

**Published:** 2021-02-09

**Authors:** Emily Bordeleau, Peter J. Stogios, Elena Evdokimova, Kalinka Koteva, Alexei Savchenko, Gerard D. Wright

**Affiliations:** a David Braley Centre for Antibiotics Discovery, M.G. DeGroote Institute for Infectious Disease Research, Department of Biochemistry and Biomedical Sciences, McMaster University, Hamilton, Ontario, Canada; b Department of Chemical Engineering and Applied Chemistry, University of Toronto, Toronto, Ontario, Canada; c Center for Structural Genomics of Infectious Diseases (CSGID), University of Calgary, Calgary, Alberta, Canada; d Department of Microbiology, Immunology and Infectious Diseases, University of Calgary, Calgary, Alberta, Canada; California State University, Fullerton; Indiana University Bloomington

**Keywords:** aminoglycoside-modifying enzymes, antibiotic resistance, apramycin

## Abstract

Apramycin is an aminoglycoside antibiotic with the potential to be developed to combat multidrug-resistant pathogens. Its unique structure evades the clinically widespread mechanisms of aminoglycoside resistance that currently compromise the efficacy of other members in this drug class. Of the aminoglycoside-modifying enzymes that chemically alter these antibiotics, only AAC(3)-IVa has been demonstrated to confer resistance to apramycin through *N*-acetylation. Knowledge of other modification mechanisms is important to successfully develop apramycin for clinical use. Here, we show that ApmA is structurally unique among the previously described aminoglycoside-modifying enzymes and capable of conferring a high level of resistance to apramycin. *In vitro* experiments indicated ApmA to be an *N*-acetyltransferase, but in contrast to AAC(3)-IVa, ApmA has a unique regiospecificity of the acetyl transfer to the N2′ position of apramycin. Crystallographic analysis of ApmA conclusively showed that this enzyme is an acetyltransferase from the left-handed β-helix protein superfamily (LβH) with a conserved active site architecture. The success of apramycin will be dependent on consideration of the impact of this potential form of clinical resistance.

## Abstract (precis)

Apramycin is an aminoglycoside antibiotic that has been traditionally used in veterinary medicine. Recently, it has become an attractive candidate to repurpose in the fight against multidrug-resistant pathogens prioritized by the World Health Organization.

## INTRODUCTION

Waksman’s tandem discoveries of streptomycin and neomycin over 65 years ago ushered in the clinical use of the aminoglycoside antibiotics (AGs) for the treatment of bacterial infections (1, 2). Since then, a variety of AGs have found clinical success. All AGs have a six-membered aminocyclitol core that serves to distinguish subfamilies of the class. For example, the 4,6-deoxstreptamine antibiotics tobramycin, gentamicin, and amikacin are particularly effective against Gram-negative pathogens. These antibiotics offer improved oto- and nephrotoxicity profiles over the 4,5-deoxstreptamine-containing scaffolds such as neomycin (3, 4). Most AGs act through noncovalent binding to the small ribosomal subunit in a fashion that disrupts the proofreading property of translation (5). The result is subversion of the genetic code followed by the production of aberrant proteins and peptides, resulting in cell death.

Soon after the introduction of AGs in clinical practice, acquired resistance mediated by mobile genetic elements was reported (6). Over the past decades, a plethora of AG resistance genes have been identified, many of them moving through bacterial populations by lateral gene transfer (7). In addition to the upregulation of efflux systems and mutations in outer membrane porins occurring in some bacteria such as Pseudomonas aeruginosa (8), two general mechanisms of resistance dominate in pathogens, drug inactivation and target modification. Both mechanisms result in a decreased affinity of the AG for its ribosomal target (9, 10). Inactivation of AGs occurs via chemical modification by one of the following three classes of aminoglycoside-modifying enzymes (AMEs): *O*-phosphorylation (APHs), *O*-adenylylation (ANTs), or *N*-acetylation by aminoglycoside acetyltransferases (AACs) (11–13). Modification of the drug target is the most recent form of aminoglycoside resistance to emerge in the clinic (14). The 16S rRNA methyltransferases (RMTases) are responsible for the N7 methylation of G1405 (e.g., Escherichia coli numbering; ArmA, Rmt family) or N1 methylation of A1408 (14, 15) (e.g., NpmA, KamB) within the 16S rRNA, respectively, conferring resistance to only 4,6-disubstituted or all 4,5- and 4,6-disubstituted AGs. RMTases are increasingly found in carbapenem-resistant isolates, greatly limiting therapeutic options for infections caused by these bacteria (16).

The broad mechanistic and genetic diversity of AG resistance impacts the use of existing drugs and dampens enthusiasm in the discovery of new members of this family of antibiotics, despite their highly desirable bactericidal activity toward Gram-positive and Gram-negative pathogens. In response to this challenge, the group at Achaogen embarked on an effort to deliver a next-generation semisynthetic AG that was not susceptible to common AMEs. The result of this effort is plazomicin (Zemdri) approved by the FDA in 2018, which retains antibiotic activity in the presence of most AMEs (17, 18). However, plazomicin remains ineffective against isolates coexpressing many RMTases, resistance genes that were not in significant circulation when the development program was launched (19). This fact emphasizes the potential for rapid dissemination of new resistance elements in the clinic that may move more rapidly through bacterial populations than the drug development process.

**FIG 1:**
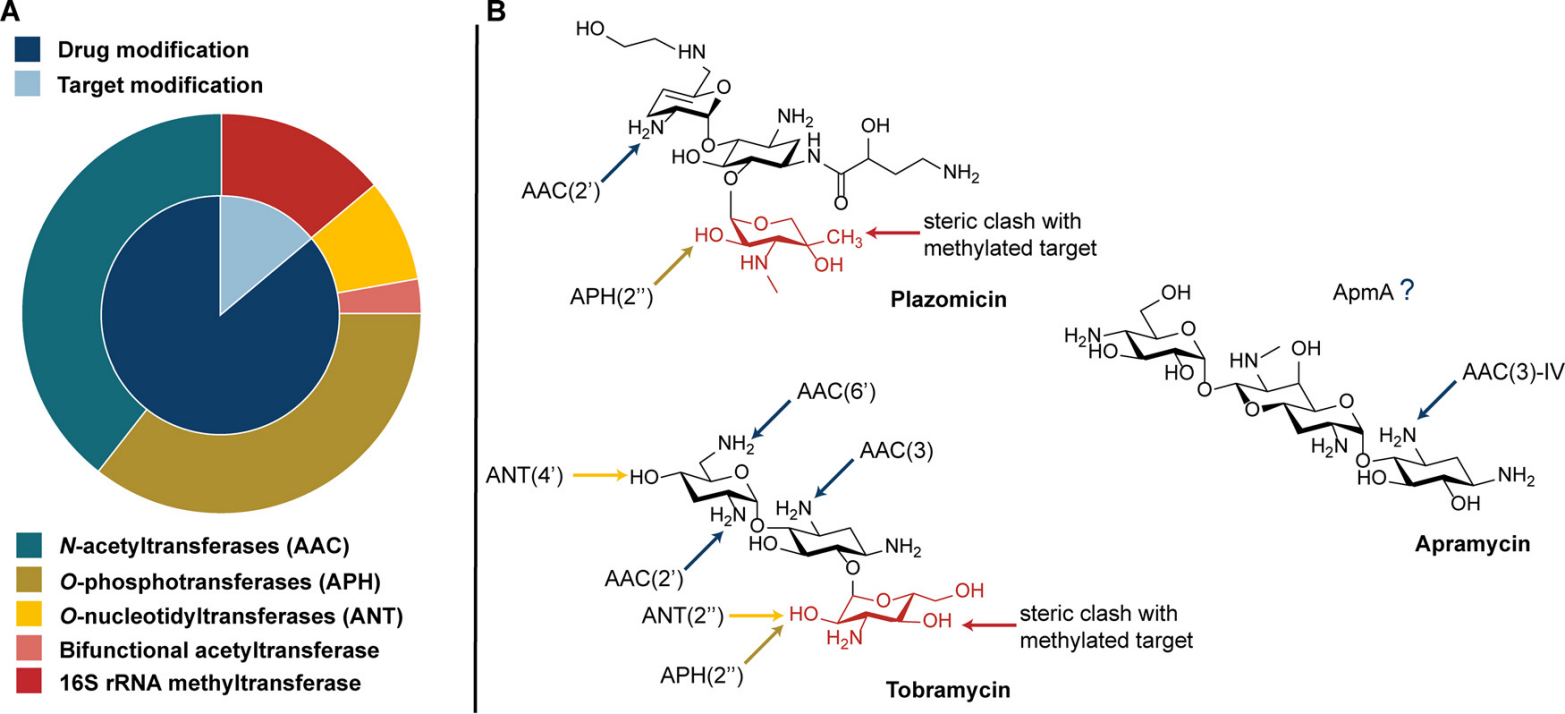
Apramycin’s advantage to overcome aminoglycoside resistance in the clinic. (A) Aminoglycoside resistance elements explored in this study (Table 1; Fig. S1). Inner circle represents the two main modes of aminoglycoside resistance, drug inactivation and target modification. Outer circle highlights the individual enzymes (Fig. S1), colored based on chemical modification made to the target or antibiotic. (B) Apramycin’s unique monosubstitution of the DOS ring with the octadiose element limits the number of inactivating mechanisms. Lack of substitution at C6 allows avoidance of clinically relevant 16S rRNA methyltransferases.

Apramycin is an unusual AG where the deoxystreptamine (DOS) aminocyclitol ring is monosubstituted and is linked to an octadiose element (Fig. 1) (20). Apramycin has been used in veterinary medicine for decades but more recently has been found to exhibit broad activity against WHO-prioritized multidrug-resistant (MDR) pathogens such as carbapenemase-producing *Enterobacteriaceae* and Acinetobacter baumannii (3, 21–24). The unique monosubstitution of apramycin’s DOS ring prevents both inactivation by a majority of common AMEs and resistance due to target alteration by N7 RMTases (24). This characteristic makes apramycin particularly attractive as a candidate next-generation AG for clinical use in humans (25).

10.1128/mBio.02705-20.1 TABLE S1 NMR assignments of acetyl-apramycin. Download Table S1, DOCX file, 0.02 MB. Copyright © 2021 Bordeleau et al. 2021 Bordeleau et al. This content is distributed under the terms of the Creative Commons Attribution 4.0 International license.

10.1128/mBio.02705-20.3 FIG S1 Apramycin resistance elements identified while screening within the aminoglycoside resistance library expressed in E. coli BW25113 Δ*tolC*Δ*bamB*. (A) Aminoglycoside resistance glycerol library layout. *N*-acetyltransferase, blue; *O*-phosphotransferase, gold; *O*-nucleotidyltransferase, yellow; bifunctional acetyltransferase, pink; 16S rRNA methyltransferase, red. Each gene was under the control of the P_bla_ promoter. (B) Control growth of library on cation-adjusted Mueller Hinton agar supplemented with no drug. Colony growth highlighted with a circle were those found to confer apramycin resistance. (C) 4 μg/ml apramycin; (D) 32 μg/ml apramycin; (E) 256 μg/ml apramycin. Download FIG S1, JPG file, 1.2 MB. Copyright © 2021 Bordeleau et al. 2021 Bordeleau et al. This content is distributed under the terms of the Creative Commons Attribution 4.0 International license.

Prior to the introduction of this antibiotic into the clinic, knowledge of the apramycin resistome is important. The *N*-acetyltransferase AAC(3)-IVa, a common selectable antibiotic resistance marker for molecular biology studies in actinomycetes, confers high-level apramycin resistance and is occasionally found in clinical isolates of *Enterobacteriaceae* (26–28). AAC(1) has been reported to be associated with apramycin resistance; however, the sequence of this gene is unavailable and the original isolates lost (A. Lovering, personal communication) (29, 30). ApmA is another acetyltransferase linked to apramycin resistance. ApmA was first reported in 2011 from bovine and porcine methicillin-resistant Staphylococcus aureus (MRSA) isolates (31, 32). The gene was found as the sole resistance element on a smaller plasmid as well as a larger antimicrobial multiresistance plasmid that also contained heavy metal resistance genes and potential virulence elements (32, 33). AAC(3)-IVa adopts the structural fold for the more recently studied AAC(3) enzymes belonging the Antibiotic_NAT family (PDB ID 6MN4). Primary sequence alignment suggests that ApmA does not share this three-dimensional structure. Given the growing interest in apramycin as a drug candidate, we have investigated ApmA’s activity toward apramycin and determined its three-dimensional structure. We identify ApmA as a member of the left-handed β-helix (LβH) superfamily, similar to chloramphenicol and streptogramin *O*-acetyltransferase resistance enzymes. This is the first report of an AG resistance element with this protein fold and reflects the diversity and enzymatic opportunism of antibiotic resistance.

## RESULTS

### The apramycin resistome is limited to four known genes.

The resistome of apramycin was evaluated through susceptibility testing against our in-house antibiotic resistance platform consisting of a panel of isogenic strains of Escherichia coli BW25113 Δ*tolC* Δ*bamB*, each expressing unique aminoglycoside resistance elements (34). Apramycin susceptibility was surveyed against 27 aminoglycoside resistance elements, consisting of 11 AACs, 11 APHs, two ANTs, and four RMTases. Gene expression levels were under the control of the constitutive promoter P_bla_. A control strain not expressing an aminoglycoside resistance element was used as a reference for apramycin potency. We found only the four previously reported apramycin resistance elements to confer resistance (Fig. S1 in the supplemental material). The two N1-A1408-directed RMTases, NpmA and KamB, and the two acetyltransferases, AAC(3)-IVa and ApmA, each confer a high level of resistance to apramycin (≥64 μg/ml; Table 1). ApmA remains the only member of these apramycin resistance elements uncharacterized with respect to its structure and function.

**TABLE 1:** Apramycin resistance elements identified through susceptibility testing of E. coli BW25113 Δ*tolC* Δ*bamB* expressing aminoglycoside resistance enzymes

Aminoglycoside resistance mechanism	Resistance gene	E. coli BW25113 Δ*tolC *Δ*bamB* MIC (μg/ml)
None	None	4
Drug modification	*aac(3)*-*IVa*	≥512
	*apmA*	64
Target modification	*kamB*	≥512
	*npmA*	≥512

### ApmA acetylates apramycin at 2′-NH_2_.

Purified recombinant ApmA was used to produce acetylated apramycin *in vitro* to determine the regiospecificity of acetyl group transfer. Characterization of the acetylated product was carried out using high-resolution electrospray ionization-mass spectrometry (HR-ESI-MS), which revealed a single acetylation of the apramycin core (mass increase of 42.0 Da) (Table 2).

**TABLE 2:** HR-ESI-MS analysis of ApmA-catalyzed acetylated aminoglycosides in positive ion mode

Modified aminoglycoside	Molecular formula	Exact mass [M+H]	
		Calculated	Observed
Apramycin	C_21_H_42_N_5_O_11_	540.2875	540.2891
*N*-acetyl-apramycin	C_23_H_45_N_5_O_12_	582.2986	582.2989

**FIG 2:**
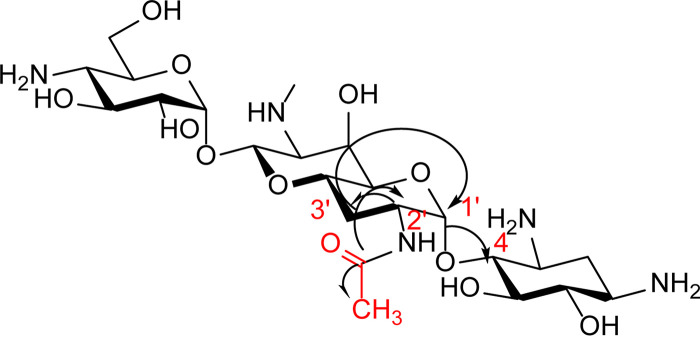
HMBC correlations. Correlations between carbonyl carbon of the acetyl group, methyl protons, and 2′ proton are indicated with the arrows.

Further characterization of the regiospecificity of acetyl transfer was accomplished using a combination of one- and two-dimensional nuclear magnetic resonance spectroscopy (1D and 2D NMR) of purified, ApmA-inactivated apramycin. (Table S1; Fig. S4 to S8) Acetyl-apramycin NMR spectra were compared with those reported in the literature for the apramycin-free base (35). We noted significant deshielding of the 2′ proton from 3.02 ppm in apramycin to 4.06 ppm in the acetylated product in the ^1^H NMR. This change is a result of the effect of a carbonyl group attached to a neighboring atom (N2′). In the ^13^C NMR, two new carbon shifts were observed at 21.8 ppm and at 172.89 ppm, corresponding to a methyl group carbon and carbonyl carbon chemical shifts. Lastly, heteronuclear multiple bond correlation spectra (HMBC) revealed correlations between the carbonyl carbon and methyl protons, as well as their correlation with the 2′ proton (Fig. 2). These data are consistent with acetylation of apramycin by ApmA at the 2′-NH_2_ of ring I.

### ApmA is an *N*-acetyltransferase from the left-handed **β**-helix protein superfamily.

Primary sequence analysis of ApmA identified a seven-hexapeptide repeat motif, suggesting it is a member of the LβH superfamily of acetyltransferases that includes the xenobiotic acetyltransferases (XAT) subclass of LβHs, responsible for the inactivation of streptogramin group A antibiotics (36, 37) (Vat) and chloramphenicol (38, 39) (CATB). Crystals of the apoenzyme and ApmA in complex with apramycin or acetyl-CoA were obtained and their structures solved to resolutions of 2.08 Å, 1.85 Å, and 2.30 Å, respectively (Table S2). Analysis of these complexes showed ApmA to be a trimeric protein and confirmed it to be a member of the LβH superfamily.

10.1128/mBio.02705-20.2 TABLE S2 X-ray diffraction data collection and refinement statistics. Download Table S2, DOCX file, 0.02 MB. Copyright © 2021 Bordeleau et al. 2021 Bordeleau et al. This content is distributed under the terms of the Creative Commons Attribution 4.0 International license.

**FIG 3:**
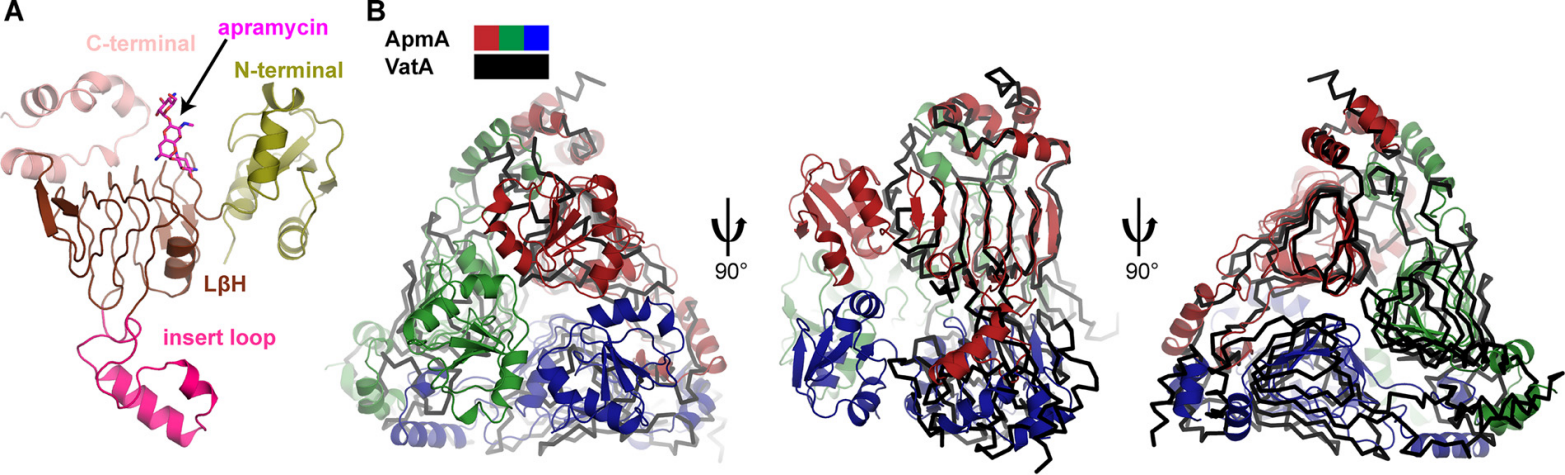
Structural comparison of ApmA to XAT subclass of LβH superfamily. (A) Domain architecture represented in ApmA monomer. (B) Structural superimposition of ApmA and VatA (PDB ID 4HUS) in complex with apramycin and virginiamycin, respectively (substrates not shown). ApmA chains are colored in red, blue, and green, and VatA chains are all colored in black.

**FIG 4:**
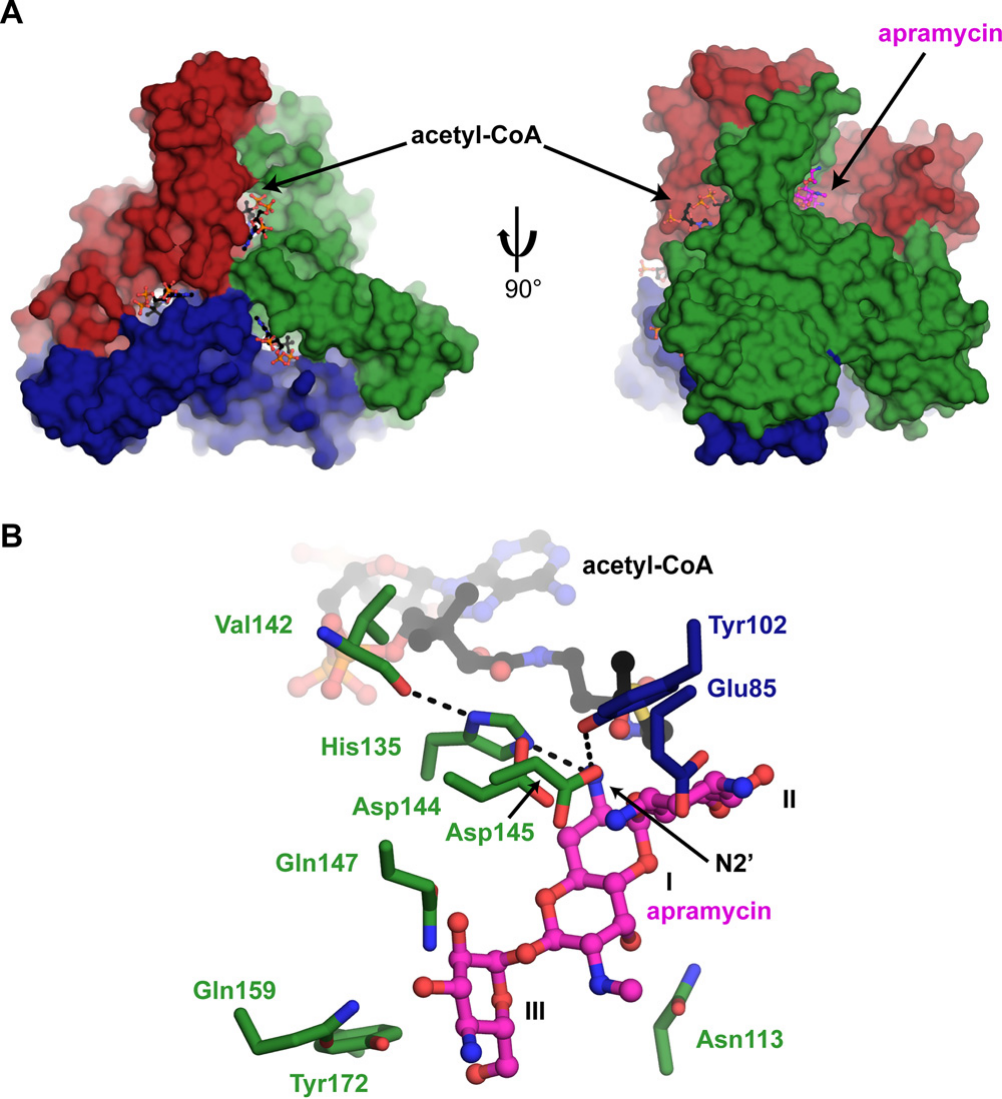
LβH domain creates a tunnel for acetyl-CoA binding. (A) Surface view of ApmA-acetyl-CoA complex superimposed on ApmA-apramycin complex. Chains are colored red, green, and blue. Apramycin and acetyl-CoA are shown in sticks. (B) Active site of superimposed ApmA substrate complexes highlighting residues suspected to be involved in apramycin binding and acetylation.

The overall structure of ApmA is consistent with the canonical XAT architecture (36): it consists of a C-terminal region comprised of three α-helices (residues 232 to 274), a central LβH domain where the hexapeptide motif is repeated seven times (residues 83 to 231), and an insert located in the center of the LβH fold, characterized by two α-helices (residues 132 to 176). Unlike other XATs, ApmA contains an additional N-terminal region, comprised of four β-sheets and two α-helices (residues 1 to 82) (Fig. 3A and B). The LβH domains of three neighboring chains together form a tunnel to shuttle the pantothenate arm of acetyl-CoA into the apramycin binding pocket (Fig. 4A). The N-terminal region appears to play a role in distorting the first two β-strands from the 7-stranded β-sheet of the LβH domain relative to their position in VatA and XAT (PDB ID 2XAT) (Fig. S2). These β-strands are twisted nearly 90° from their position in VatA and XAT and significantly alter the shape and volume of the substrate binding pocket (Fig. S2).

10.1128/mBio.02705-20.4 FIG S2 Impact of the N-terminal region on the substrate binding pocket of ApmA. Red arrows indicate the first two β-strands from the 7-stranded β-sheet of the LβH domain. Superimpositions of ApmA in complex with apramycin (A), CAT (B), and VatA (C). Download FIG S2, JPG file, 0.3 MB. Copyright © 2021 Bordeleau et al. 2021 Bordeleau et al. This content is distributed under the terms of the Creative Commons Attribution 4.0 International license.

**FIG 5:**
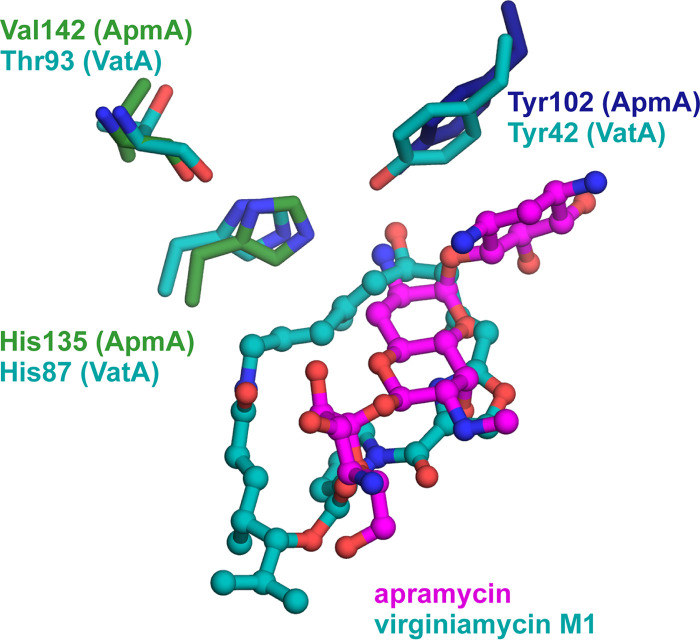
Superimposition of active site for VatA (PDB ID 4HUS) onto ApmA. Important catalytic residues for VatA and the equivalent residues found in ApmA are identified.

Three acetyl-acceptor binding pockets were identified with the positioning of the central DOS ring consistent with other AMEs, which typically are lined with aspartate and glutamate residues (40). In the ApmA-apramycin complex, the N3 atom of the AG’s aminocyclitol ring is coordinated by two aspartic acids (Asp144 and Asp145) in a helix within the C-terminal domain and one glutamic acid (Glu85) position the N1 atom. Ring III of apramycin interacts with several residues from one of the helices of the inserted loop region of the LβH domain (Gln147, Gln159, and Tyr172). The 6′-OH of ring I interacts with N113 from within the LβH domain (Fig. 4A and 4B). Consistent with the NMR structure of 2′-acetyl-apramycin, we observed the 2′-NH_2_ of the antibiotic positioned for acetylation. Upon superimposition of the two binary complexes (root mean square deviation [RMSD], 0.29 Å), the 2′-NH_2_ lies between 3.1 to 3.7 Å from the carbonyl carbon of the acetyl moiety (Fig. 4B). Tyr102 participates in a network of hydrogen bonds between the 2′-NH_2_ and Asp144 that may be important for antibiotic binding. The imidazole side chain from His135 is within 2.8 to 3.1 Å of apramycin’s 2′-NH_2_. Consistent with other XAT enzymes, the N1 of this imidazole is hydrogen bonded to a carbonyl of the peptide backbone (Val142) (Fig. 4B). It is suspected that interaction increases the basicity of imidazole’s N2 to help in a role as a general base (37, 38). Superimposition of ApmA with VatA, in complex with their antibiotic substrates, demonstrates that the His135 of ApmA is geometrically equivalent to the catalytic histidine for VatA (His87) (36) (Fig. 5). Tyr102 of ApmA was also found in the same position of Tyr42 of VatA, a residue responsible for stabilizing the oxyanion intermediate for *O*-acetylation (36). To further assess the significance of His135 at the sequence level, we gathered reference sequences for Vats and CATBs from the Comprehensive Antibiotic Resistance Database (CARD) (41) to construct a multiple sequence alignment. Our analysis revealed that His135 of ApmA aligns with the conserved catalytic histidine found across all Vat and CATB sequences, essential for the *O*-acetylation of their respective substrates (36) (Fig. S3). We next generated the alanine mutant of His135 to evaluate the impact of this substitution on ApmA’s detoxification of apramycin through cell-based assays. Upon expression of the His135Ala mutant in E. coli, resistance to apramycin remained within 2-fold of when the wild-type enzyme was expressed (32 to 64 μg/ml). These results suggest the role of this histidine in the acetylation of apramycin does not hold the same significance as for the function of other XATs and requires further investigation.

10.1128/mBio.02705-20.5 FIG S3 Sequence relationship of ApmA to the LβH superfamily. Full multiple sequence alignment of ApmA with CATs and Vats obtained from the Comprehensive Antibiotic Resistance Database. Amino acids are colored based on percent identity. Traditional hexapeptide motif (*i*, *i *+ 1, and *i *+ 4) of the LβH domain is highlighted in red. Conserved catalytic histidine of the XAT subclass is highlighted in pink. Download FIG S3, JPG file, 2.8 MB. Copyright © 2021 Bordeleau et al. 2021 Bordeleau et al. This content is distributed under the terms of the Creative Commons Attribution 4.0 International license.

10.1128/mBio.02705-20.6 FIG S4 ^1^H NMR for 2′-acetyl-apramycin. Download FIG S4, PDF file, 0.1 MB. Copyright © 2021 Bordeleau et al. 2021 Bordeleau et al. This content is distributed under the terms of the Creative Commons Attribution 4.0 International license.

## DISCUSSION

**FIG 6:**
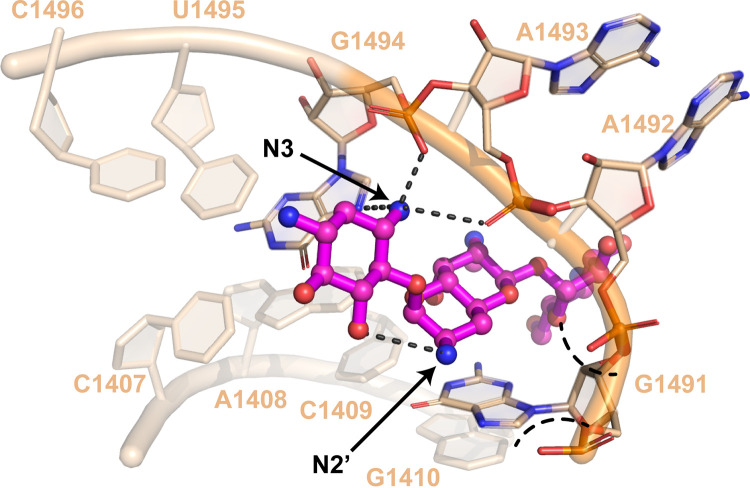
Impact of 2′ acetylation on ribosomal binding of apramycin. Crystal structure of apramycin bound to the ribosome (PDB ID 4AQY). Important interactions between N3 and the ribosome for recognition of the 2-DOS ring are indicated. Intramolecular interaction between N2′ and O5 is highlighted. Acetylation at position N3 creates steric clash with the phosphate backbone. Acetylation of the N2′ position is predicted to create an electronic clash with the negatively charged backbone of A1492 and G1491.

Apramycin’s atypical structure in comparison with other AGs has garnered considerable attention for its potential as a next-generation AG antibiotic. The development of derivatives, termed apralogs, has focused on retaining apramycin’s low ototoxic potential while increasing potency coupled with evading resistance to apramycin through AAC(3)-IV-mediated modification (42, 43). Molecular modeling of 3-acetyl-apramycin bound to the ribosome indicates that reduced binding to the target results from a steric clash between the 16S rRNA phosphate backbone and the amide positioned at C3 of apramycin (24). We show that ApmA is an acetyltransferase that instead modifies apramycin at the N2′ position of the octadiose element to confer high-level drug resistance. However, N2′ does not make direct contacts with the 16S rRNA and could spatially accommodate the acetyl group. We suggest that acetylation at this position reduces the affinity of the overall molecule for its target. The N2′ participates in an intramolecular interaction with O5 of the 2-DOS ring. Intramolecular interactions between AG sugar rings have been suggested to play an important role in the recognition and binding of AGs to the 16S rRNA (44). The octadiose element of apramycin participates in important hydrogen bonds with A1408 of the 16S rRNA, creating a glycoside-adenine pseudo-base pair (43, 45, 46). Acetylation of N2′ would disrupt the orientation of the 2-DOS and octadiose ring. The carbonyl would also have the potential to create new intramolecular interactions that alter the configuration of the molecule. Unfavorable intermolecular interactions would also be introduced if the carbonyl group approaches the negatively charged phosphate backbone, creating an electronic clash. Lastly, the overall charge of apramycin will be impacted by the replacement of the primary amine with an amide. The removal of the positively charged amine would further hinder its ability to interact with the negatively charged RNA (Fig. 6).

The regiospecificity of the acetyl transfer phenotypically assigns ApmA to the AAC(2′) family of AMEs. However, unlike ApmA, AAC(2′) enzymes are members of the GNAT superfamily (19) and are found chromosomally encoded in Providencia stuartii and Mycobacterium tuberculosis (47, 48). Our analysis of AG resistance shows that AAC(2′)-Ia from P. stuartii does not confer resistance toward apramycin, making ApmA the first AAC(2′) enzyme documented to do so. Furthermore, initial reports of *apmA* found the sequence mobilized on plasmids from *Staphylococci* isolates from bovine and swine (31–33). The most recent report of the gene has also identified *apmA* in *Campylobacter* isolated from pork, demonstrating an expansion of host and crossover to Gram-negative pathogens (49).

The findings of this study highlight the adaptation of a common protein fold to generate new functions in antibiotic resistance. Concerns that other XAT enzymes capable of conferring resistance to other classes of antibiotics were discussed over 20 years ago (50). Antibiotic acetyltransferases are products of convergence of function, with CoA-dependent acetylation spanning several protein scaffolds (39, 51–55). Our crystallographic data demonstrate ApmA belongs to the LβH superfamily of acetyltransferases, which had not previously been linked to the detoxification of AG antibiotics. The substrate specificity of the LβH scaffold has expanded to accommodate AGs, reminiscent of other sugar-containing substrates of LβH *N*-acetyltransferases involved in *O*-antigen biosynthesis (56, 57). Previously, XATs were observed to only *O*-acetylate and their respective antibiotic substrates with the help of a conserved catalytic histidine. There are also LβH *N*-acetyltransferases that contain a histidine in the active site and have been shown to be important in the acetylation of their respective nucleotide-linked sugar substrates (56, 58). We show that the His135Ala ApmA mutant is still capable of *N*-acetylating apramycin to confer an equivalent level of resistance to when the wild-type protein is expressed. This suggests that the molecular mechanism of acetyl transfer is similar to that of the GCN5 family AAC(6′) enzymes where no catalytic base is necessary. Here, acyl transfer occurs due to the alignment and proximity of acetyl-CoA and the nucleophilic receptor amine of the antibiotic (59).

Overall, this work has the potential to aid in the identification of apralogs with reduced susceptibility to ApmA. Furthermore, the distribution of *apmA* should be monitored for further transfer into clinical isolates. This unique AME threatens not only the success of apramycin’s introduction into the clinic but may impact other AGs susceptible to its modification as well.

## MATERIALS AND METHODS

### Bacterial strains and *apmA* cloning.

*apmA* (GenBank accession no. FN806789.3) was synthesized as a gBlock by Integrated DNA Technologies (IDT) for cloning into pGDP3 (34) and pET19b-TEV using NdeI and XhoI restriction sites. The pET19b-TEV plasmid consists of an N-terminal His_10_ tag cleavable by a tobacco etch virus protease (TEV). The pGDP3 construct of *apmA* was transformed into hyperpermeable, efflux-deficient mutant E. coli BW25113 Δ*tolC* Δ*bamB* (for antimicrobial susceptibility testing). Construct of pET19b-TEV:*apmA* was transformed into BL21(DE3)-Gold competent cells (for crystallography). All constructs were verified through Sanger sequencing at the Mobix sequencing facility, McMaster University.

### Site-directed mutagenesis.

Nucleotide substitutions (indicated by bold text in primers) were introduced in *apmA* with PCR site-directed mutagenesis by primer extension (60) using pGDP3:*apmA* as a template and the following mutagenic oligonucleotide primers to produce *apmA*:H135A: forward, 5′-AGAGATCCATGCGAAC**GCT**CAGTTAAACATGACCTTTG-3′, and reverse, 5′-AAGGTCATGTTTAACTG**AGC**GTTCGCATGGATCTCTG-3′.

The final construct was verified through Sanger sequencing at the Mobix sequencing facility, McMaster University.

### Antimicrobial susceptibility testing.

Screening against our in-house antibiotic resistance platform was carried out as previously described (34). Apramycin susceptibility testing of E. coli BW25113 Δ*tolC* Δ*bamB* expressing *apmA* and *apmA*(H135A) was completed in triplicate following the Clinical and Laboratory Standards Institute (CLSI) protocols for the broth microdilution method (61). Strains were cultured in cation-adjusted Mueller-Hinton broth (CAMHB) in a 96-well format. Plates were incubated in a shaking incubator at 37°C for 18 h.

### Protein expression and purification.

For acetylated product characterization, E. coli BL21(DE3) Rosetta-gami pLysS transformed with pET19b-TEV constructs of *apmA* were grown in autoinduction medium supplemented with selection antibiotics for 3 days at 25°C and 180 rpm. Cells were collected by centrifugation at 6,400 × *g*, 4°C, and resuspended in binding buffer (25 mM HEPES [pH 7.5], 300 mM NaCl, and 10 mM imidazole). Resuspended cells were lysed using a continuous cell disrupter at 20,000 lb/in^2^, 4°C, followed by centrifugation at 30,000 × *g* to remove cell debris. ApmA was purified from the lysate by nickel-nitrilotriacetic acid (Ni-NTA) affinity chromatography (Qiagen) at 4°C. A 2-ml volume of Ni-NTA resin was preequilibrated with binding buffer and incubated with the lysate for 1 h on ice prior to purification. The resin was washed with wash buffer (25 mM HEPES [pH 7.5], 300 mM NaCl, and 25 mM imidazole), and proteins were eluted with a 4-stepwise gradient (25%, 50%, 75%, and 100%) of elution buffer (25 mM HEPES [pH 7.5], 300 mM NaCl, and 250 mM imidazole). Elutions were dialyzed overnight against dialysis buffer (25 mM HEPES [pH 7.5], 300 mM NaCl). SDS-PAGE gel electrophoresis was performed to assess sample purity. To prepare stock solutions, concentrated ApmA was diluted to a final concentration ranging from 30 μM to 150 μM in dialysis buffer. Protein dilutions were flash frozen in liquid nitrogen and stored at −80°C.

For crystallography studies, native ApmA was expressed in Gold competent E. coli BL21(DE3). A 3-ml overnight culture was diluted into 1 liter of LB media containing selection antibiotics and grown at 37°C with shaking. Expression of selenomethionine-derivatized ApmA was carried out in M9 minimal media following the manufacturer’s instructions (Shanghai Medicilon). Expression was induced with isopropyl β-d-1-thiogalactopyranoside (IPTG) at 17°C when the optical density at 600 nm (OD_600_) reached 0.6 to 0.8. The overnight cell culture was then collected by centrifugation at 7,000 × *g*. Cells were resuspended in binding buffer (100 mM HEPES [pH 7.5], 500 mM NaCl, 5 mM imidazole, and 5% glycerol [vol/vol]) and lysed with a sonicator, and cell debris was removed by centrifugation at 30,000 × *g*. The cell lysate was loaded on a 4-ml Ni-NTA column (Qiagen) preequilibrated with 250 ml of binding buffer. The resin was washed with wash buffer (100 mM HEPES [pH 7.5], 500 mM NaCl, 30 mM imidazole, and 5% glycerol [vol/vol]), and the proteins were eluted with elution buffer (100 mM HEPES [pH 7.5], 500 mM NaCl, 250 mM imidazole, and 5% glycerol [vol/vol]). The His_10_-tagged proteins were then subjected to overnight TEV cleavage using 50 μg of TEV protease per mg of His_10_-tagged protein in binding buffer and dialyzed overnight against the binding buffer. The His_10_-tag and TEV were removed by running the protein again over the Ni-NTA column. The tag-free proteins were dialyzed against dialysis buffer (50 mM HEPES [pH 7.5], 500 mM NaCl) overnight, and the purity of the protein was analyzed by SDS-polyacrylamide gel electrophoresis.

### Spectroscopic characterization of ApmA-catalyzed acetylated apramycin.

ApmA-catalyzed acetylated apramycin was produced from 50-ml *in vitro* reactions (50 mM HEPES, pH 7.5) consisting of 500 μM aminoglycoside, 500 μM acetyl-CoA, and 1 μM ApmA. Reaction mixtures were incubated at room temperature until acetylated products (mass increase of 42.0 Da) were detected by liquid chromatography (LC)/ESI-MS. Enzymes were removed by centrifugation using an Amicon Ultra-15 centrifugal filter and the flowthrough subsequently concentrated. Acetyl-apramycin was purified from the concentrate using AG50W-X8 strong cation resin. The resin was preequilibrated with 1% NH_4_OH and washed with H_2_O until a neutral pH was obtained. Fractions containing acetylated products were identified by LC/ESI-MS, followed by detailed analysis with NMR and HR-ESI-MS. LC/ESI-MS data were acquired using a QTrap 2000 (Applied Biosystems) system equipped with an Agilent 1100 LC interface. HR-ESI-MS data were acquired using an Agilent 1290 ultraperformance liquid chromatography (UPLC) separation module and quadrupole time of flight (Q-TOF) G6550A mass detector in positive ion mode. NMR analysis was completed using a Bruker AVIII 700 MHz instrument in deuterated water as the solvent. The chemical shifts are reported in parts per million.

### Crystallization and structure determination.

All crystals were grown at room temperature using the vapor diffusion sitting drop method with 0.5 μl of protein solution mixed with 0.5 μl of reservoir solution. Crystals were grown using the following reservoir solutions: ApmA plus apramycin complex (0.2 M CaCl, 20% polyethylene glycol 3350 [PEG 3350], and 5 mM apramycin) and ApmA plus acetyl-CoA complex (0.1 M citric acid, pH 3.6, 30% PEG 200, 5 mM apramycin, and 2 mM acetyl-CoA). All crystals were cryoprotected with Paratone-N or ethylene glycol and then flash frozen in liquid nitrogen prior to diffraction data collection. Diffraction data were collected at the Advanced Photon Source, Argonne National Laboratory, beamlines 19-ID or 21-ID. All data were processed by HKL-3000. The ApmA plus apramycin structure was solved using the single anomalous diffraction (SAD) method, and this was used to solve all additional complexes by MR. Structure refinement was performed using Phenix.refine plus manual building with Coot. The presence of substrate molecules was identified by building into the Fo-Fc difference density after the initial rounds of refinement.

### Sequence and structural analysis.

PyMOL (62) was used to identify potential interacting residues (≤4.0 Å in distance) of ApmA with substrates apramycin and acetyl-CoA. Structural superimpositions with VatA (PDB ID 4HUS) were constructed using the cealign function in PyMol. XAT representative sequences were obtained from the CARD (41). ApmA and Vat sequences were aligned with the Expresso (63–65) function of T-Coffee (66, 67) to build a profile hidden Markov model (HMMER 3.3.1) (68). All sequences were aligned with the resulting HMM profile and visualized using Jalview (69).

### Data availability.

PDB validation reports for crystal structures obtained in this study have been submitted with the manuscript. Accession numbers are as follows: 7JM0 (ApmA apoenzyme), 7JM1 (ApmA complex with acetyl-CoA), and 7J6M2 (ApmA complex with apramycin).

10.1128/mBio.02705-20.7 FIG S5 ^13^C NMR for 2′-acetyl-apramycin. Download FIG S5, PDF file, 0.1 MB. Copyright © 2021 Bordeleau et al. 2021 Bordeleau et al. This content is distributed under the terms of the Creative Commons Attribution 4.0 International license.

10.1128/mBio.02705-20.8 FIG S6 ^1^H-^1^H correlation spectroscopy (COSY) NMR for 2′-acetyl-apramycin. Download FIG S6, PDF file, 0.1 MB. Copyright © 2021 Bordeleau et al. 2021 Bordeleau et al. This content is distributed under the terms of the Creative Commons Attribution 4.0 International license.

10.1128/mBio.02705-20.9 FIG S7 ^1^H-^13^C heteronuclear single quantum coherence (HSQC) for 2′-acetyl-apramycin. Download FIG S7, PDF file, 0.1 MB. Copyright © 2021 Bordeleau et al. 2021 Bordeleau et al. This content is distributed under the terms of the Creative Commons Attribution 4.0 International license.

10.1128/mBio.02705-20.10 FIG S8 ^1^H-^13^C HMBC for 2′-acetyl-apramycin. Download FIG S8, PDF file, 0.1 MB. Copyright © 2021 Bordeleau et al. 2021 Bordeleau et al. This content is distributed under the terms of the Creative Commons Attribution 4.0 International license.
