# Supplementary material for: ApmA Is a Unique Aminoglycoside Antibiotic Acetyltransferase That Inactivates Apramycin
Source: mBio. 2021 Feb 9;12(1):e02705-20. doi: 10.1128/mBio.02705-20 (PMC7885111; doi:10.1128/mBio.02705-20)
Supplement: TABLE S1 [file mBio.02705-20-st001.docx]

**Table S1. NMR assignments of acetyl-apramycin**

| Carbon number | Apramycin standard | | Acetyl-apramycin | |
| --- | --- | --- | --- | --- |
|  | ^1^H (ppm)(35) | ^13^C (ppm) | ^1^H (ppm) | ^13^C (ppm) |
| 1 | 2.74 | 50.2 | 2.79 (m, 1H) | 50.24 |
| 2 | 1.23  2.00 | 35.4 | 1.26 (m, 1H)  2.04 (m, 1H) | 34.57w |
| 3 | 2.87 | 49.3 | 2.88 (m, 1H) | 49.60 |
| 4 | 3.31 | 86.7 | 3.34 (t, *J* = 9.4 Hz, 1H) | 87.19 |
| 5 | 3.49 | 75.9 | 3.52 (m, 1H) | 75.78 |
| 6 | 3.16 | 77.3 | 3.19 (t, *J* = 9.6 Hz, 1H) | 77.33 |
| 1’ | 5.16 | 100.6 | 5.18 (d, *J* = 3.6 Hz, 1H) | 98.30 |
| **2’** | **3.02** | 48.9 | **4.06 (dt, *J* = 12.7, 4.1 Hz, 1H)** | 48.01 |
| **2’**-NH-CO-**CH_3_** | NA | NA | **2.00 (s, 3H)** | 21.8 |
| **2’**-NH-**CO**-CH_3_ | NA | NA | NA | 172.89 (HMBC) |
| **3’** | **1.67**  **2.12** | 31.8 | **1.82 (q, *J* = 11.8 Hz, 1H) 2.17**  **2.17 (m, 1H)** | 29.52 |
| 4’ | 3.78 | 67.1 | 3.84 (m, 1H) | 66.65 |
| 5’ | 3.67 | 70.1 | 3.68 (m, 1H) | 72.95 |
| 6’ | 4.28 | 65.3 | 4.31 (m, 1H) | 65.33 |
| 7’ | 2.68 | 61.4 | 2.72 (m, 1H) | 61.56 |
| 8’ | 4.92 | 95.5 | 4.96 (d, *J* = 8.5 Hz, 1H) | 95.77 |
| 1” | 5.37 | 94.5 | 5.38 (d, *J* = 2.5 Hz, 1H) | 94.38 |
| 2” | 3.59 | 70.8 | 3.57 (m, 1H) | 72.0 |
| 3” | 3.65 | 73.3 | 3.63 (m, 1H) | 73.91 |
| 4” | 2.74 | 52.2 | 2.76 (m, 1H) | 52.14 |
| 5” | 3.65 | 72.5 | 3.66 (m, 1H) | 73.91 |
| 6” | 3.72  3.85 | 60.9 | 3.78 (m,1H)  3.75 (m, 1H) | 60.35 |
| N-Me | 2.38 | 32.0 | 2.40 | 32.0 |
