# Supplementary figures and images for: ApmA Is a Unique Aminoglycoside Antibiotic Acetyltransferase That Inactivates Apramycin
Source: mBio. 2021 Feb 9;12(1):e02705-20. doi: 10.1128/mBio.02705-20 (PMC7885111; doi:10.1128/mBio.02705-20)

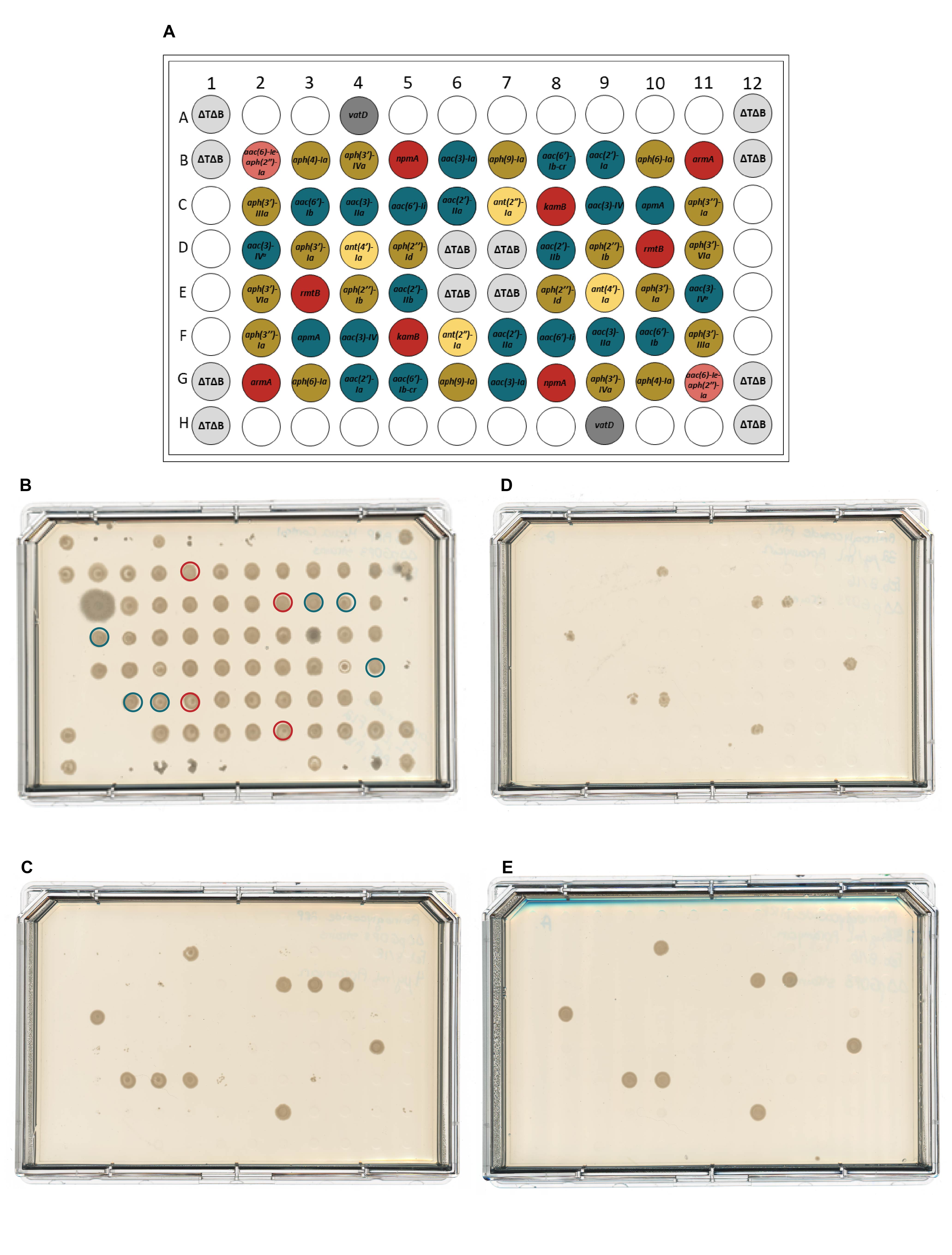

Supplement: FIG S1 [file mBio.02705-20-sf001.jpg]

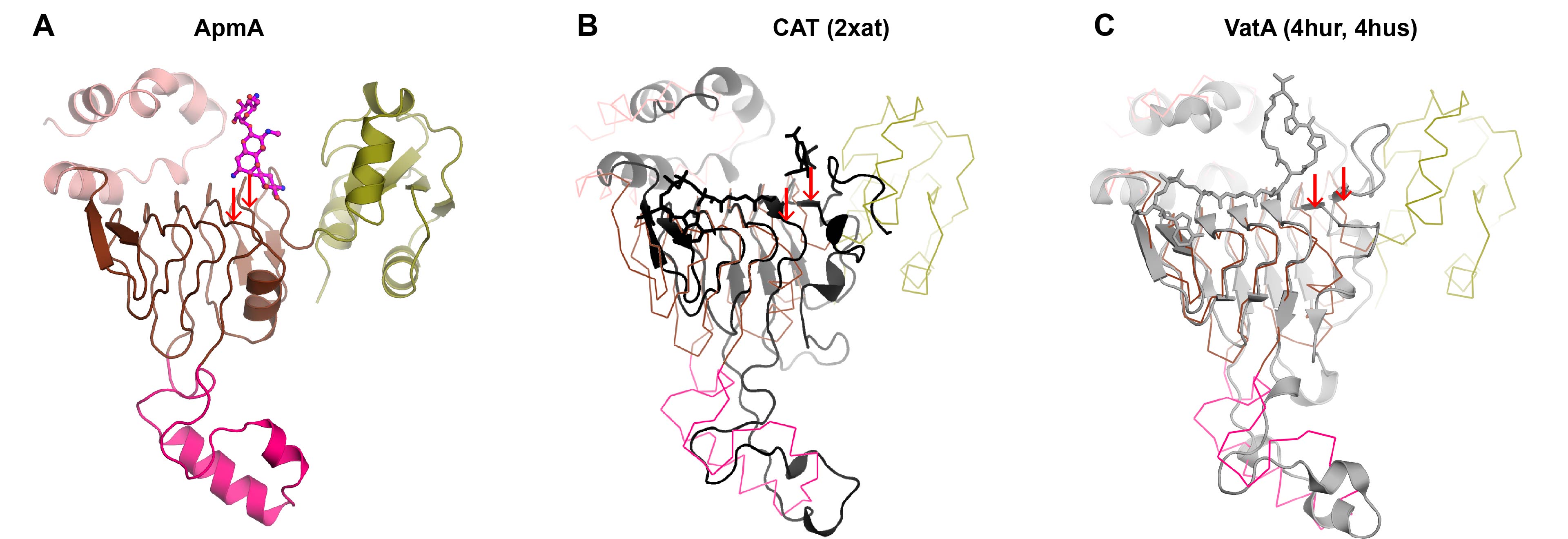

Supplement: FIG S2 [file mBio.02705-20-sf002.jpg]

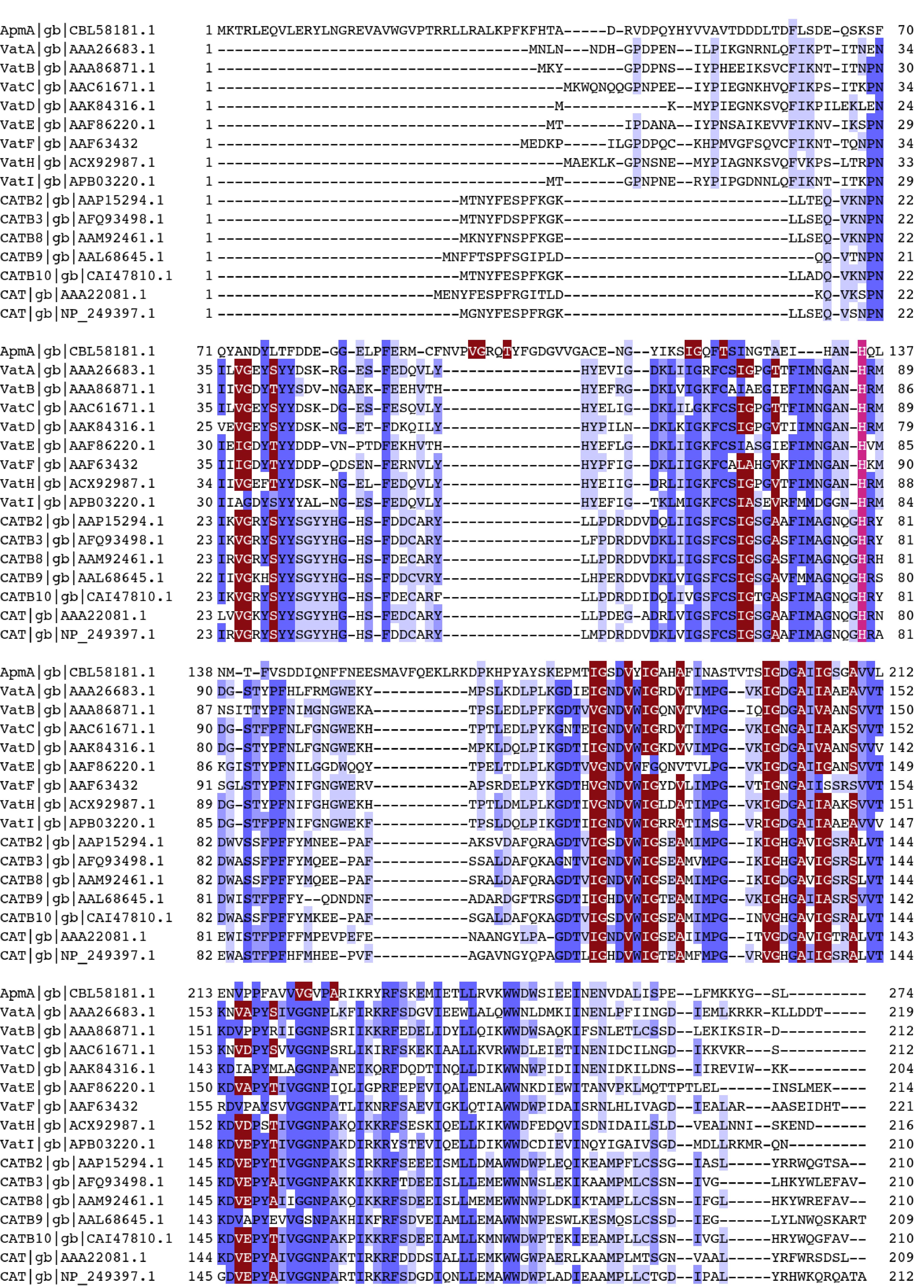

Supplement: FIG S3 [file mBio.02705-20-sf003.jpg]

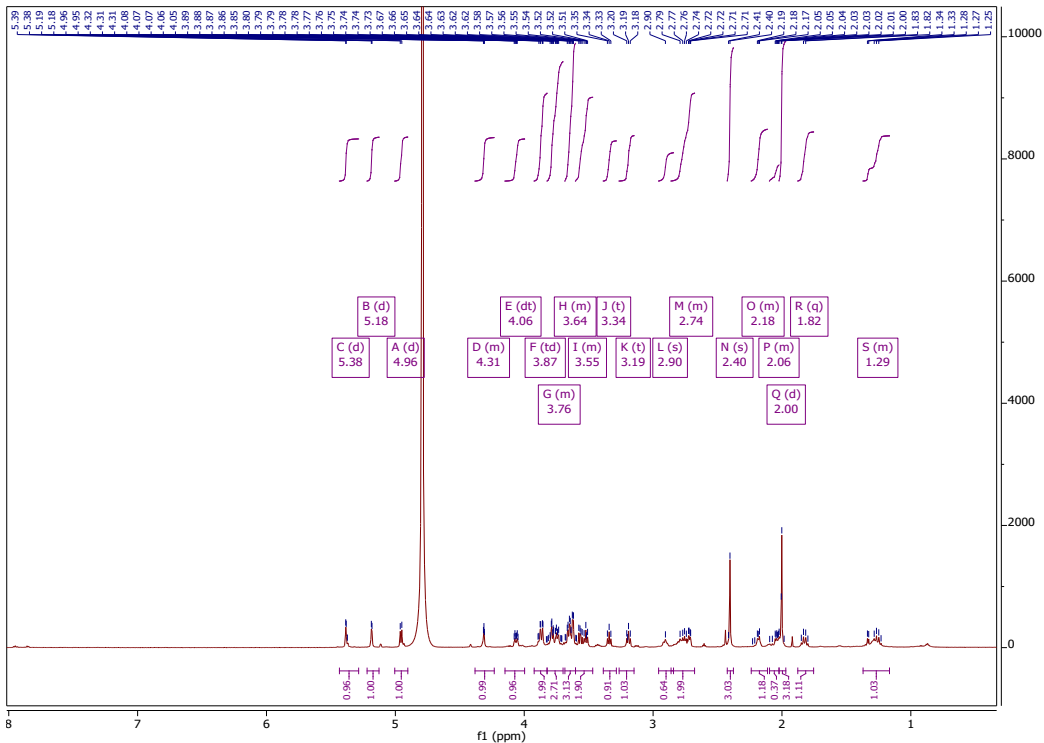

Supplement: FIG S4 [file mBio.02705-20-sf004.pdf]

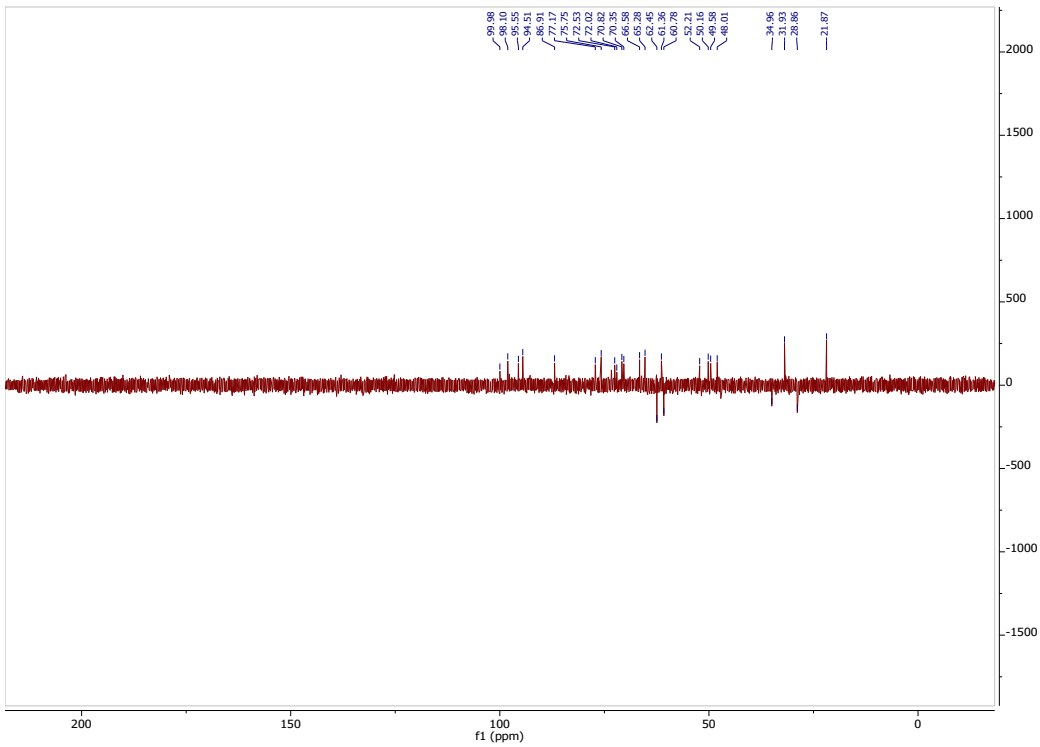

Supplement: FIG S5 [file mBio.02705-20-sf005.pdf]

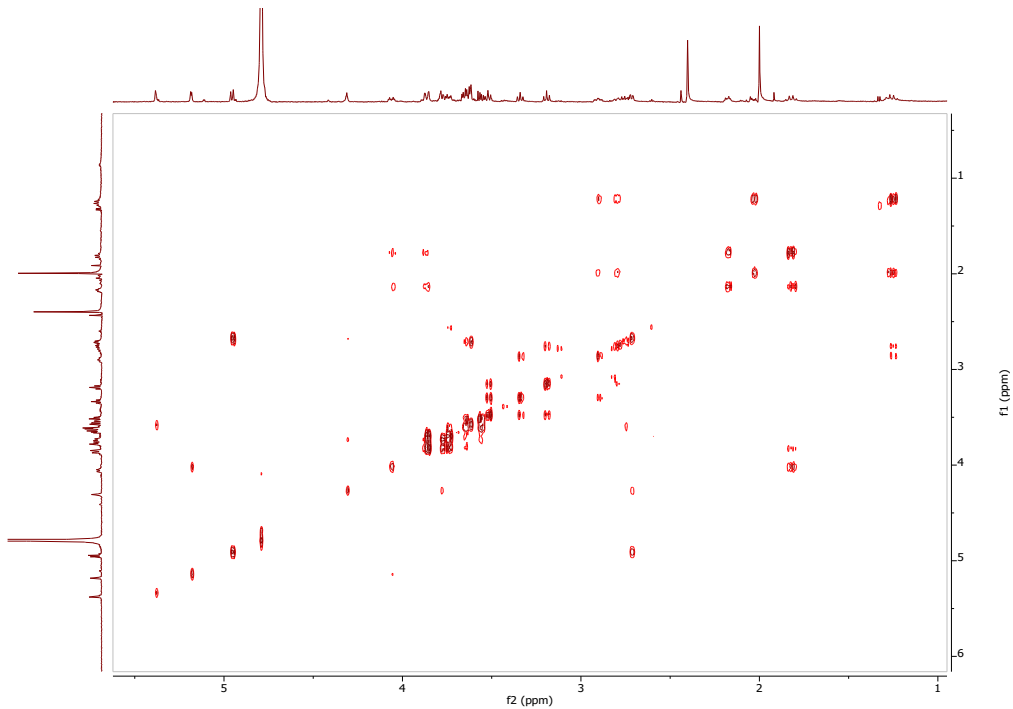

Supplement: FIG S6 [file mBio.02705-20-sf006.pdf]

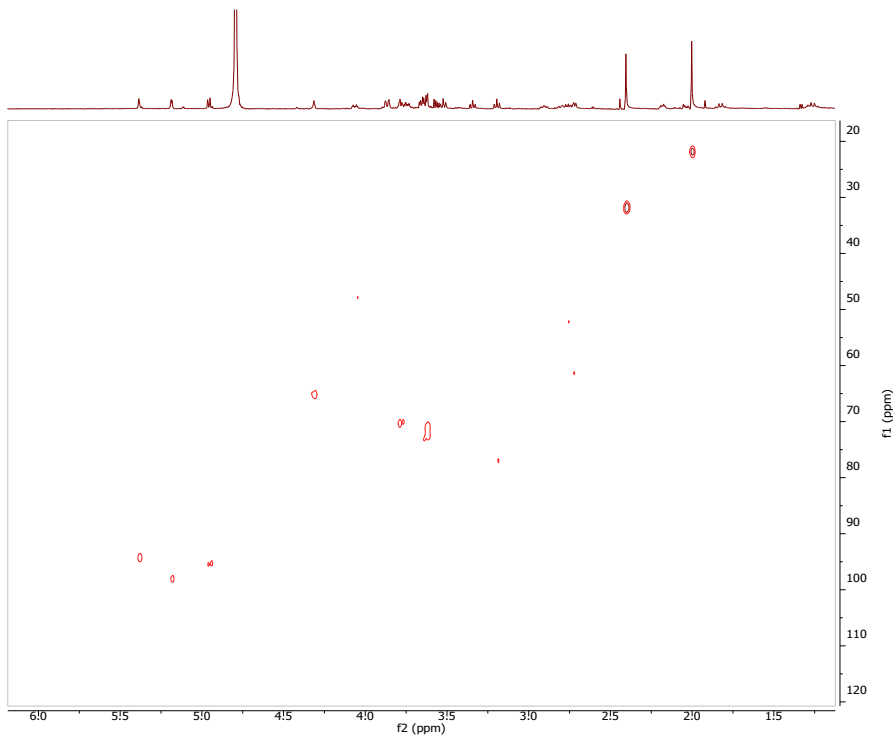

Supplement: FIG S7 [file mBio.02705-20-sf007.pdf]

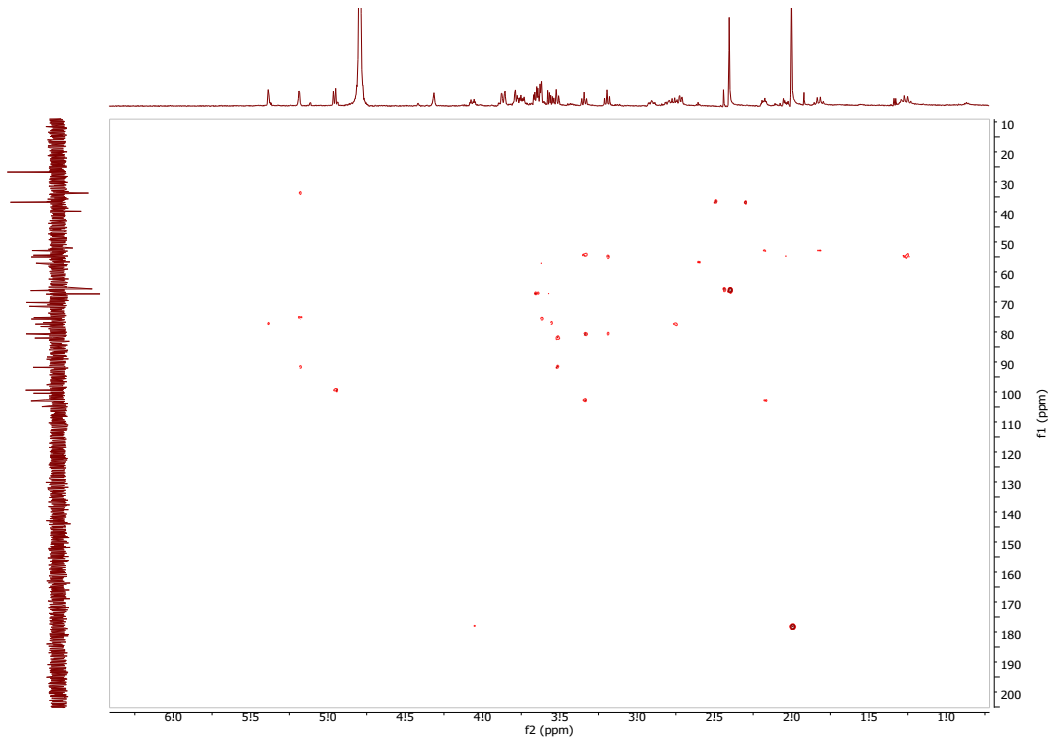

Supplement: FIG S8 [file mBio.02705-20-sf008.pdf]
