# Supplementary material for: ApmA Is a Unique Aminoglycoside Antibiotic Acetyltransferase That Inactivates Apramycin
Source: mBio. 2021 Feb 9;12(1):e02705-20. doi: 10.1128/mBio.02705-20 (PMC7885111; doi:10.1128/mBio.02705-20)
Supplement: TABLE S2 [file mBio.02705-20-st002.docx]

**Table S2. X-ray diffraction data collection and refinement statistics.**

| Structure | ApmA apoenzyme | ApmA•acetyl-CoA complex | ApmA•apramycin complex |
| --- | --- | --- | --- |
| PDB code | 7JM0 | 7JM1 | 7JM2 |
| Data collection |  |  |  |
| Space group | P2_1_ | P2_1_ | P2_1_2_1_2_1_ |
| Cell dimensions  *a*, *b, c* (Å)  α, β, γ, (°) | 68.54, 76.92, 96.57  90, 90, 90 | 68.20, 77.23, 97.44  90, 90, 90 | 61.73, 107.54, 138.70  90, 90, 90 |
| Resolution, Å | 25.00 – 2.08 | 40.00 – 2.30 | 40.00 – 1.85 |
| R*_merge_^a^*  R*_pim_* | 0.109 (0.451)  0.075 (0.297) | 0.041 (0.120)  0.027 (0.083) | 0.068 (0.691)^b^  0.041 (0.459) |
| *I* / σ(*I)* | 19.38 (2.08) | 19.3 (7.5) | 22.60 (2.17) |
| Completeness, % | 98.7 (100) | 97.5 (96.3) | 97.3 (94.5) |
| Redundancy | 3.2 (3.2) | 3.0 (2.8) | 3.6 (3.1) |
|  |  |  |  |
| Refinement |  |  |  |
| Resolution, Å | 25.00 – 2.08 | 37.81 – 2.30 | 38.92 – 1.85 |
| No. of unique reflections:  working, test | 58230, 2000 | 42297, 3597 | 76382, 1971 |
| *R*-factor/free *R­*-factor^c^ | 18.8/22.2 (22.0/27.1) | 15.6/19.7 (17.7/24.2) | 19.7/23.6 (40.6/39.5) |
| No. of refined atoms, molecules  Protein  Apramycin  Acetyl-CoA  Solvent  Water | 6597, 3  N/A  N/A  5  609 | 6565, 3  N/A  153, 3  N/A  686 | 6572, 3  111, 3  N/A  2  769 |
| *B*-factors  Protein  Apramycin  Acetyl-CoA  Solvent  Water | 35.3  N/A  N/A  76.8  43.0 | 33.9  N/A  65.6  N/A  47.4 | 35.7  28.4  N/A  57.0  42.91 |
| r.m.s.d.  Bond lengths, Å  Bond angles, ° | 0.002  0.526 | 0.014  1.463 | 0.006  0.779 |

^a^*R*_sym_ = Σ_h_Σ_i_|*I*_i_(*h*) - 〈*I*(h)〉/Σ_h_Σ_i_I_i_(*h*), where *I*_i_(*h*) and 〈*I*(*h*)〉 are the *i*th and mean measurement of the intensity of reflection *h*.

^b^Figures in parentheses indicate the values for the outer shells of the data.

^c^*R* = Σ|F_p_^obs^ – F_p_^calc^|/ΣF_p_^obs^, where F_p_^obs^ and F_p_^calc^ are the observed and calculated structure factor amplitudes, respectively.

* = molecules in the active site cleft.
